# Nanoparticles with High-Surface Negative-Charge Density Disturb the Metabolism of Low-Density Lipoprotein in Cells

**DOI:** 10.3390/ijms19092790

**Published:** 2018-09-17

**Authors:** Xue Bai, Jiaxin Zhang, Ya-Nan Chang, Weihong Gu, Runhong Lei, Yanxia Qin, Shibo Xia, Sihan Ma, Yuelan Liang, Kui Chen, Juan Li, Baoyun Sun, Gengmei Xing

**Affiliations:** 1 CAS Key Laboratory for Biomedical Effects of Nanomaterial and Nanosafety, Institute of High Energy Physics, Chinese Academy of Science (CAS), Beijing 100049, China; xbai@ihep.ac.cn (X.B.); zhangjiaxin@ihep.ac.cn (J.Z.); changyn@ihep.ac.cn (Y.-N.C.); guwh@ihep.ac.cn (W.G.); leirh@ihep.ac.cn (R.L.); qinyx@ihep.ac.cn (Y.Q.); xiasb@ihep.ac.cn (S.X.); mash@ihep.ac.cn (S.M.); liangyl@ihep.ac.cn (Y.L.); chenkui@ihep.ac.cn (K.C.); sunby@ihep.ac.cn (B.S.); 2 University of Chinese Academy of Sciences (UCAS), Beijing 100049, China

**Keywords:** low-density lipoprotein, endocytosis, gold particles, surface charge density

## Abstract

Endocytosis is an important pathway to regulate the metabolism of low-density lipoprotein (LDL) in cells. At the same time, engineering nanoparticles (ENPs) enter the cell through endocytosis in biomedical applications. Therefore, a crucial question is whether the nanoparticles involved in endocytosis could impact the natural metabolism of LDL in cells. In this study, we fabricated a series of gold nanoparticles (AuNPs) (13.00 ± 0.69 nm) with varied surface charge densities. The internalized AuNPs with high-surface negative-charge densities (HSNCD) significantly reduced LDL uptake in HepG-2, HeLa, and SMMC-7721 cells compared with those cells in control group. Notably, the significant reduction of LDL uptake in cells correlates with the reduction of LDL receptors (LDL-R) on the cell surface, but there is no change in protein and mRNA of LDL-Rs. The cyclic utilization of LDL-R in cells is a crucial pathway to maintain the homoeostasis of LDL uptake. The release of LDL-Rs from LDL/LDL-R complexes in endosomes depended on reduction of the pH in the lumen. AuNPs with HSNCD hampered vacuolar-type H^+^-ATPase V1 (ATPaseV1) and ATPaseV0 binding on the endosome membrane, blocking protons to enter the endosome by the pump. Hence, fewer freed LDL-Rs were transported into recycling endosomes (REs) to be returned to cell surface for reuse, reducing the LDL uptake of cells by receptor-mediated endocytosis. The restrained LDL-Rs in the LDL/LDL-R complex were degraded in lysosomes.

## 1. Introduction

Endocytosis is an important pathway to regulate the metabolism of biomolecules in cells [1]. The engineered nanoparticles (ENPs) enter cells through endocytosis [2,3], which makes it indispensable to determine their impact on intrinsic metabolism of the cell.

In general, the metabolism of extracellular macromolecules in cells is firstly recognized by the corresponding receptor on the cell membrane and dissociated from plasma membrane through a coated vesicle [4,5]; the coated vesicle fuses with early endosomes (EEs) and release the cargos [6]; the cargos are sorted in the endosome, and are then transported to lysosome for degradation [7]. low-density-lipoprotein receptor (LDL-R) system has become recognized as a prototype of the process of receptor-mediated endocytosis [1], which coordinates the metabolism of cholesterol, an essential component of the plasma membrane of all mammalian cells [8]. Moreover, in the metabolism of LDL by the endocytosis pathway, freed receptors from the LDL/LDL-R complex in endosome enter recycling endosomes (REs) for reuse [9]. The defective genes’ encoding of LDL-R proteins [10] or disruption of LDL-R recycling in cells [11] results in accumulation of LDL in serum.

Numerous studies have confirmed that negative surface charge improves the biocompatibility of the ENPs and enhances the advantage of application in biological medicine [9]. Engineered gold nanoparticles (AuNPs) have been used as drug deliverers [12], image agents [13], and to treat drugs [14]. Due to AuNPs’ tremendous potential in medical applications, their absorption [15], distribution [16], and excretion [17,18] in cells have been researched. Endocytosis of small-size nanoparticles was through pinocytosis that accompanies the endocytosis of extracellular molecules binding on receptors [19]. Nuri Oh et al. suggest that the AuNP exocytosis pattern was not significantly governed by the size, and nanoparticles cleared in macrophage could be modulated by engineering their surface chemistry [17]. However, one crucial question is whether the surface charge of AuNPs impacts the natural metabolism of extracellular molecular in cells by the endocytosis pathway.

Here, we fabricated a series of small-size AuNPs (13.00 ± 0.69 nm) with varied surface negative-charge densities to investigate the impact of these engineered AuNPs on the metabolism of LDL in cells.

## 2. Results

### 2.1. Characterization of AuNPs

**Figure 1:**
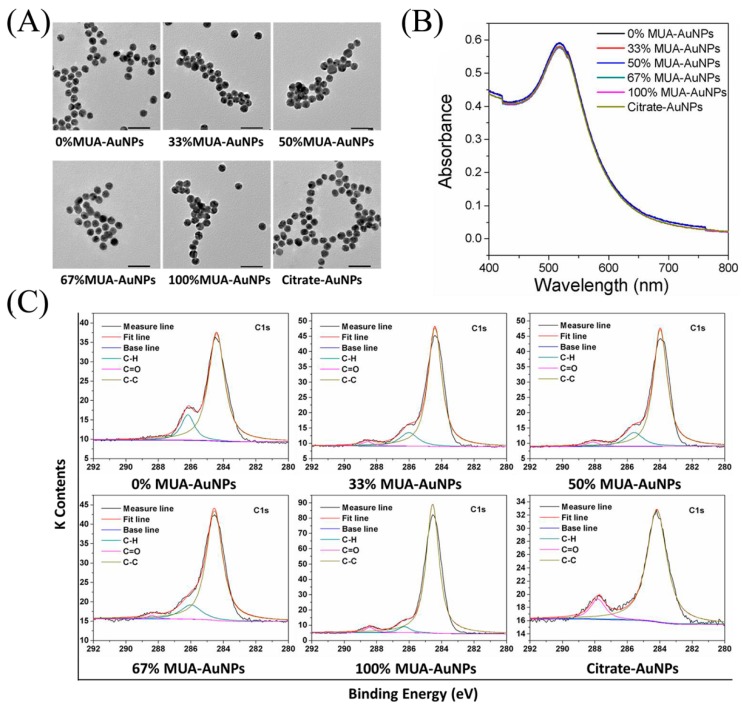
Characterization of gold nanoparticles (AuNPs). (**A**) Transmission electron microscopy (TEM) images and (**B**) ultraviolet (UV)-vis absorption of AuNPs with different surface negative-charge density. (**C**) X-ray photoelectron spectroscopy (XPS) spectra of C1s. Bar = 50 nm.

In the present study, we regulated the surface negative-charge densities of AuNPs through arranging mixed self-assembled monolayers containing 11-mercaptoundecanoic acid (MUA) and 1-octanethiol (OT). Transmission electron microscopy (TEM) images of AuNPs showed that the particles had sizes of 13.00 ± 0.69 nm and were spherical in shape (Figure 1A). In addition, dynamic light scattering (DLS) revealed that the hydrodynamic size of these AuNPs in culture medium was around 42 nm, with no significant differences. Furthermore, the zeta potentials of 100% MUA–AuNPs and citrate-AuNPs were −33.14 ± 0.42 and −32.05 ± 1.77 mV, respectively, which were significantly higher than those of other AuNPs (Table 1). The characteristic local-surface plasmon resonance of these AuNPs at 520 nm did not show spectrum peak broadening, implying that all nanoparticles were homogeneously dispersed (Figure 1B). Moreover, the X-ray photoelectron spectroscopy (XPS) spectrum demonstrated varied surface chemical groups on these AuNPs (Figure 1C) and the formation of the Au–S bond (Figure S1).

After being incubated with the AuNPs for 24 h at 37 °C, the amount of AuNPs taken up by cells was measured using inductively coupled plasma mass spectrometry. The results showed that the amount of AuNPs taken up by cells was higher for 100% MUA–AuNPs and citrate–AuNPs than 0%, 33%, 50%, and 67% MUA–AuNPs (Figure S2).

### 2.2. Effects of AuNPs on LDL Uptake in Cells

Varied cell viability following treatment with AuNPs was assayed; a concentration of 3 μg/mL for AuNPs did not induce changes in cell viability or increase the apoptosis ratio (Figure S3), which dose will be used for further experiments.

**Figure 2:**
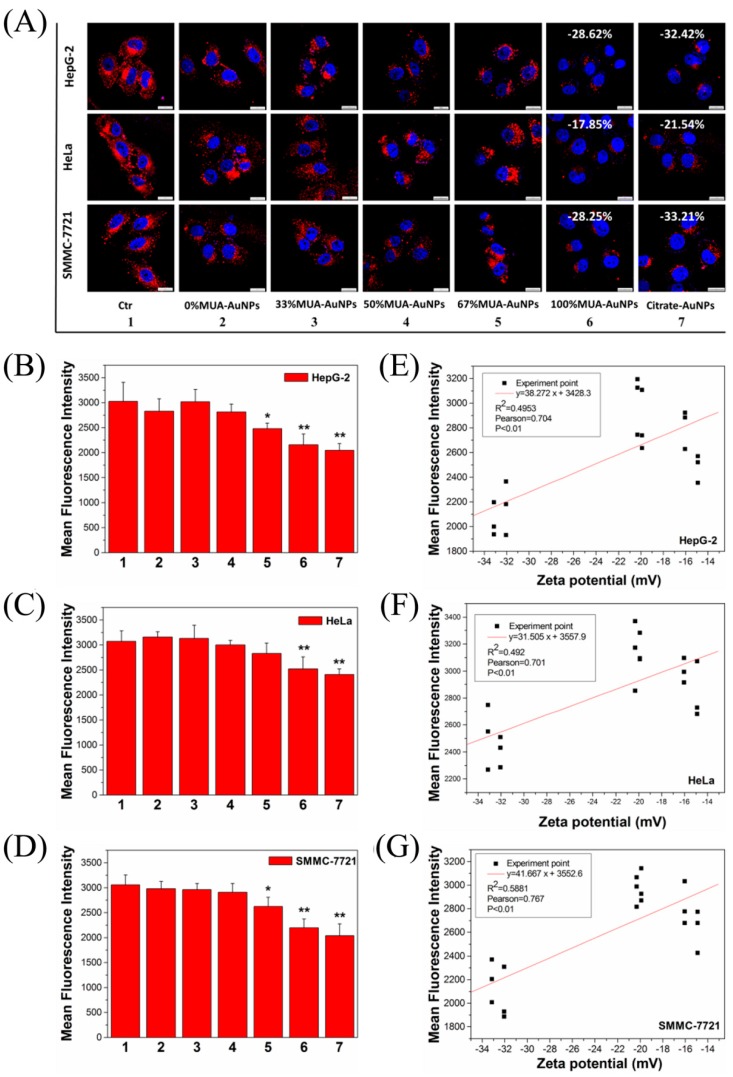
Effects of AuNPs on 1,1′-Dioctadecyl-3,3,3′,3′-tetramethylindocarbocyanine (DiI-LDL) uptake in cells. (**A**) The cells were treated with 10 μg/mL DiI-LDL for 2 h at 37 °C and observed by laser-scanning confocal microscopy (LSCM). (**B**–**D**) Mean fluorescence intensity (MFI) for the uptake of DiI-LDL was determined using flow cytometry (FCM) after 2 h at 37 °C. The correlation analysis between the zeta potential and the MFI of DiI-LDL for (**E**) HepG-2 cells, (**F**) HeLa cells, and (**G**) SMMC-7721 cells. Subfigures 1–7 represent the cells in the control group and cells with 0% MUA–AuNPs, 33% MUA–AuNPs, 50% MUA–AuNPs, 67% MUA–AuNPs, 100% MUA–AuNPs, and citrate–AuNPs, respectively. The data for MFI represent the means ± SEM from our three independent experiments. ** *p* < 0.01, * *p* < 0.05 represent significant differences compared with the control group. Bar = 20 μm.

Laser-scanning confocal microscopy (LSCM) images tracked the internalization of LDL in cells and showed the red fluorescence intensity of LDL with the label of 1,1′-Dioctadecyl-3,3,3′,3′-tetramethylindocarbocyanine (DiI-LDL) in the cells. After exposure to 100% MUA–AuNPs and citrate–AuNPs, the fluorescence intensity decreased in various human endothelial cells (HepG-2, HeLa, and SMMC-7721 cells) compared with that in control cells, whereas others AuNPs did not show reduced fluorescence intensity (Figure 2A). Compared with the control group, the statistical data from flow cytometry (FCM) analysis indicated that the mean fluorescence intensity (MFI) of DiI-LDL in cells decreased by about 28.62% and 32.42% in HepG-2 cells (Figure 2B), 17.85% and 21.54% in HeLa cells (Figure S2C), and approximately 28.25% and 33.21% (Figure 2D) in SMMC-7721 cells, respectively, following treatment with 100% MUA–AuNPs and citrate–AuNPs. Furthermore, a significant negative correlation between the MFI of DiI-LDL in cells and the surface zeta potential of AuNPs was observed in HepG-2, HeLa, and SMMC-7721 cells (*p* < 0.01, Figure 2E–G); there was no significant difference following treatment with 100% MUA–AuNPs and citrate–AuNPs. In a subsequent experiment, we chose the two nanoparticles (100% MUA–AuNPs and citrate–AuNPs) to treat HepG-2 cells for investigating the reason of reduction of LDL uptake.

### 2.3. Effects of AuNPs on LDL Binding to LDL-R on the Cell Surface

**Figure 3:**
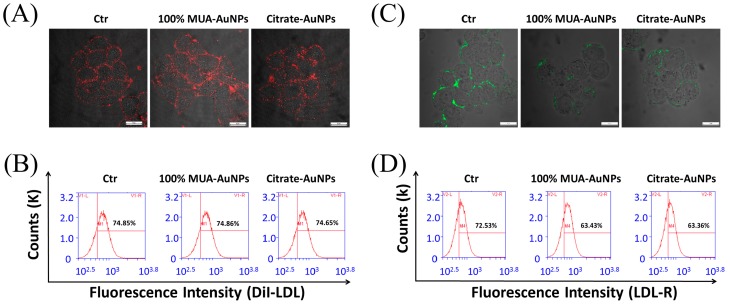
Effects of AuNPs on DiI-LDL binding to LDL-R on the cell surfaces. HepG-2 cells were incubated with AuNPs at 4 °C for 20 min, and then incubated with 10 μg/mL DiI-LDL at 4 °C for 20 min. Cells were then (**A**) observed by LSCM at a magnification of 400×, and the MFI of DiI-LDL was measured (**B**) using FCM. HepG-2 cells were cultured with AuNPs at 37 °C for 24 h, incubated with anti-LDL-R antibodies with fluorescein isothiocyanate at 4 °C for 20 min, and observed under LSCM at a magnification of 400× (**C**). The MFI of LDL-R (**D**) was measured by FCM.

A reduced LDL amount in cells suggests that the metabolism of LDL through endocytosis pathway was adjusted by AuNPs with HSNCD. We examined whether the AuNPs occupied the binding sites of LDL at LDL-R, thereby hampering the uptake of LDL by receptor-mediated endocytosis. HepG-2 cells were coincubated with AuNPs at 4 °C for 20 min, and the medium was then replaced with a fresh medium; DiI-LDL with red fluorescence was introduced, and cells were cultured again for 20 min at 4 °C. DiI-LDL was removed, and the cells were observed with LSCM. The obtained images showed that the fluorescence intensity was not different between the treated and control cells (Figure 3A). In addition, the statistical results of the FCM analysis supported this observation (Figure 3B). Based on these observations, we concluded that the AuNPs did not occupy binding sites to inhibit the binding between LDL and LDL-R on the cell surface.

Furthermore, we observed and assayed LDL-R on the surface of the HepG-2 cells using anti-LDL-R antibodies after the cells were coincubated with AuNPs for 24 h at 37 °C. The images from LSCM showed that 100% MUA–AuNPs and citrate–AuNPs decreased the fluorescence intensity of the antibody compared with that in the control group (Figure 3C), indicating that LDL-R on the cell surface was reduced by treatment with AuNPs for 24 h. In particular, the statistical data from FCM analysis showed that 100% MUA–AuNPs and citrate–AuNPs decreased the fluorescence intensity of LDL-R by about 12.48% and 13.76%, respectively, on HepG-2 cell surfaces compared with that of the control group (Figure 3D). These results imply that fewer LDL-Rs on the cell surface reduced LDL binding, subsequently affecting the internalization of LDL by endocytosis.

### 2.4. Effects of AuNPs on LDL-R Gene Expression

**Figure 4:**
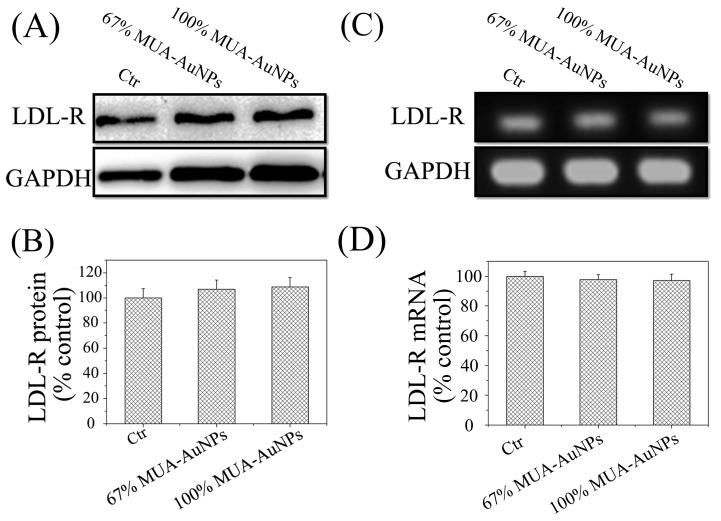
Effects of AuNPs on LDL-R expression. (**A**) A representative blot of LDL-R protein and (**B**) the normalized intensity of LDL-R versus GAPDH. Reverse transcription-PCR (RT-PCR) was performed with primers targeting LDL-R and GAPDH (**C**), and the level of LDL-R mRNA was measured using quantitative RT-PCR (**D**). The means ± SEM from three independent experiments were evaluated.

Next, we evaluated whether the AuNPs could alter the gene expression of LDL-R at the mRNA and protein levels. A representative LDL-R blot, as shown in Figure 4A, was quantitatively analyzed based on average gray values. The three test groups in the quantitative analysis of LDL-R protein in HepG-2 cells treated for 24 h, as in the previous case, showed no significant differences (Figure 4B). These results of reverse transcription-PCR (RT-PCR) (Figure 4C) and quantitative real-time PCR (q-RT-PCR) (Figure 4D) also supported that *LDL-R* gene expression in cells was not modulated by the AuNPs. This observation indicated that the AuNPs affected the distribution of LDL-R on the cell surface through a pathway independent of *LDL-R* gene expression.

### 2.5. Effects of AuNPs on LDL Transport in Cells

**Figure 5:**
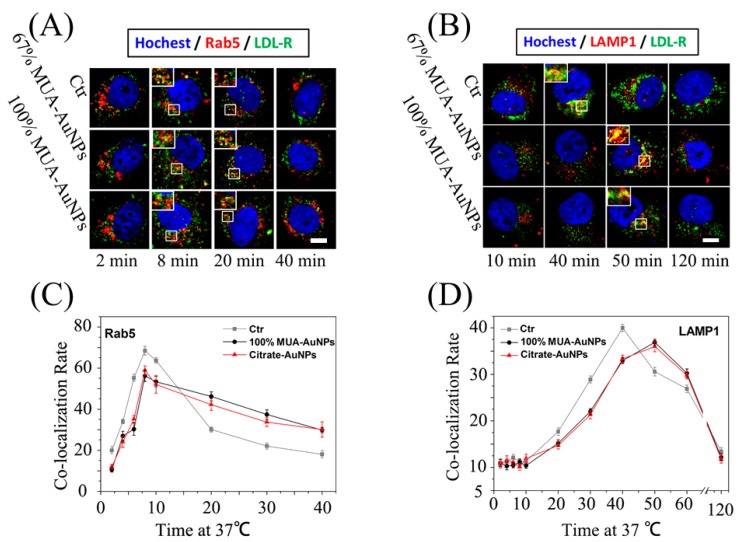
Effects of AuNPs on the LDL transport rate in cells. After treatment, HepG-2 cells were precooled at 4 °C for 10 min, followed by replacement of the medium, and subcultured in medium with 10% lipoprotein-deficient fetal bovine serum (LPDS) and 10 μg/mL DiI-LDL at 4 °C for 20 min. Next, the unbound DiI-LDL was washed, and the cells were shifted and recultured at 37 °C for the required time. The colocalization between LDL and (**A**) Rab5 or (**B**) LAMP1 was observed using LSCM. The colocalization ratios were measured using NIS-Elements for (**C**) Rab5 and (**D**) LAMP1. The colocalization ratios are shown as the means ± SEM from three independent experiments; more than 50 cells were selected for every experiment. Bar = 5 μm.

The vesicle transport of internalized LDL by endocytosis in cells was tracked using fluorescent colocalization of LDL and marker proteins of vesicles. The colocalization rate was measured as the percentage of LDL (red) pixel values above background. In Figure 5A, the fluorescent colocalization (yellow spots) of LDL and EEs (marked by Ras-related protein-5, Rab5) showed that LDL was located at the EEs both in control and treated cells at 8 min; furthermore, the yellow spots reduced the duration from 8 to 20 min in the control cells, but this reduction was not observed in the treated cells. Statistical data showed that the colocalization rate of LDL and EEs in treated cells was higher than that in control cells at 20 min (Figure 5C). Moreover, even at 40 min, the yellow spots were still visible in the treated cells, but were uncommon in the control cells (Figure 5A,C). Under normal conditions, by endocytosis, the internalized LDL was eventually degraded in the lysosome, and the peak of colocalization of LDL and lysosomes (marked by lysosomal-associated membrane protein 1, LAMP1) was observed at 40 min in control cells, as mostly internalized LDL was transported to the lysosomes. However, in treated cells, the colocalization spots appeared even at 50 min (Figure 5B,D), indicating that AuNPs hampered the vesicle transport of LDL from EEs to lysosomes in cells and that LDL was thus arrested in the EEs. These results imply that the LDL/LDL-R complex was trapped in EEs by the AuNPs.

In the endosomes, the LDL is dissociated from the LDL/LDL-R complex and then is transported in lysosomes to degrade, and free receptors are returned to the plasma membrane through the REs for reuse. LSCM was used to observe the colocalization of LDL-R with REs (marked by Rab11). The colocalization rate of LDL-R in REs was lower in the treated cells for 24h than in the control cells (Figure S4C), implying that LDL-R was arrested in EEs and was not transported to REs. Statistical analysis of the colocalization rate in cells in the fluorescence images using NIS-Elements supported this observation (Figure S4D). We therefore suggest that the release of LDL-R from LDL/LDL-R complex was hampered by the AuNPs with HSNCD.

### 2.6. Retained LDL-R Was Degraded in Lysosomes

**Figure 6:**
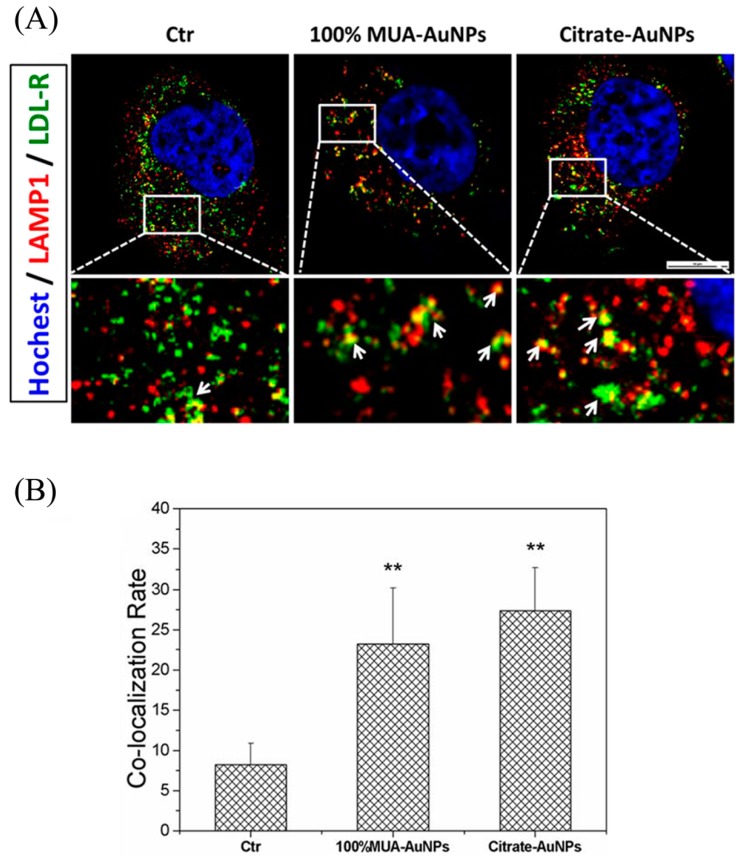
Retained LDL-R in early endosomes (EEs) transported to the lysosome for degradation. Fluorescence images were captured using LSCM (**A**), and quantitative data were analyzed using NIS-Elements, white arrows indicate the colocalization area between EEs and LDL-R. (**B**). The colocalization ratios were presented as the means ± SEM from three independent experiments, and more than 30 cells were selected for each experiment. ** *p* < 0.01 indicates significant differences in the treated cells compared with the control group. Bar = 10 μm.

As demonstrated by previous studies, LDL-R mutations blocking the release of LDL prevent receptor recycling, leading to LDL-R degradation in lysosomes [20]. Thus, we observed and detected the colocalization of LDL-R and lysosomes (marked by LAMP1) in cells to determine whether the retained LDL-R in EEs was degraded in lysosomes. The colocalization rate was measured as the percentage of LDL-R (green) pixel values above background. As shown in Figure 6A, the colocalization area (yellow spots) of LDL-R and lysosomes in the treated cells was larger than that in control cells. In addition, the statistical data revealed that the colocalization rate of LDL-R and lysosomes was about 8% in control cells and increased significantly, to around 23% and 27%, in cells treated with 100% MUA–AuNPs and citrate–AuNPs (Figure 6B). These results implied that the LDL/LDL-R complex arrested at EEs owing to AuNP treatment was transported to the lysosomes for degradation.

### 2.7. AuNPs Disrupted pH Regulation in Endosomes

**Figure 7:**
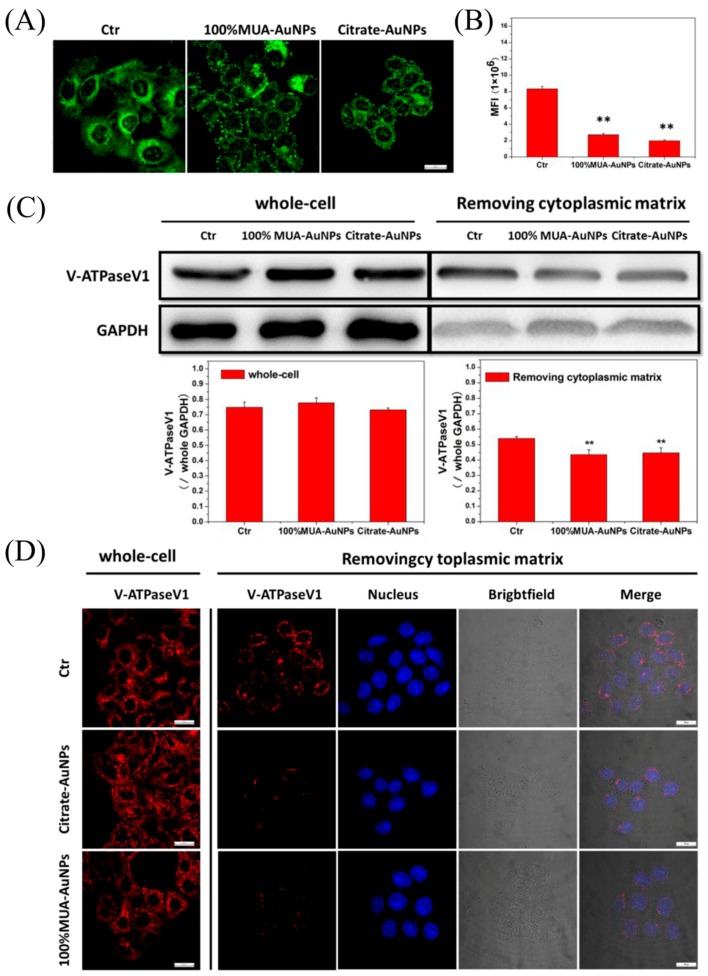
The acidic regulation rate was decreased by AuNPs in cell endosomes. LysoSensor Green DND-189 was coincubated with cells at 37 °C for 2 min, and replaced the medium. (**A**) Observations were made with LSCM; (**B**) 50 cells were selected to compute single-cell MFI for every experiment. (**C**) The extract of the whole cells and the cells without cytoplasmic were used for western blot assay of V-ATPaseV1; (**D**) immunofluorescence image of V-ATPaseV1 for whole cells and the cells without cytoplasmic, which were attained by incubating with 0.003% digitonin at 4 °C for 15 min. The histogram data represents the mean ± SEM values from three independent experiments. ** *p* < 0.01 represents significant differences in the treated cells compared with the control group. Bar = 10 μm.

A reduction in the pH value induces the release of LDL from the LDL/LDL-R complex. Thus, we observed variations in pH in the acidic endosome (pH 5.5–5.0) in control and treated cells using LysoSensor Green DND, whose pKa was ~5.2. The fluorescent intensity of the LysoSensor increased with an increasingly acidic environment. LSCM images showed that the green fluorescence intensity in the treated cells was weaker than that in control cells (Figure 7A). We then randomly selected 50 cells to compute the MFI of a single cell. Our results showed that the MFI of a single cell in the treated group was significantly lower than that in the control group (Figure 7B). These results indicated that compared with the control cells, the endosomes in treated cells were less acidic.

The pH change in endosomes depended on the activity of V-ATPase, which, in turn, depended on the binding of V-ATPaseV1 and V-ATPaseV0 [21]. V-ATPaseV1 was localized in the cytoplasm, and V-ATPaseV0 was localized on the plasma membranes of the vesicle. Therefore, we determined the binding of V-ATPaseV1 on the plasma membrane of vesicles using western blotting. The gray value of V-ATPaseV1 protein from whole cells did not differ significantly between the treated cells and control cells, whereas the gray value in transported vesicles of treated cells was lower than that in control cells; the statistical analysis showed that the rates of reduction were about 19.59% and 17.50% for 100% MUA–AuNPs and citrate–AuNPs, respectively, compared with that in control cells (Figure 7C). Immunofluorescence assays of V-ATPaseV1 also supported these results. As shown in Figure 7D, V-ATPaseV1 showed a scattered distribution throughout the cell for the control and treated groups, and there were no obvious differences in the fluorescence intensity of V-ATPaseV. After removing the cytoplasmic matrix from the cell as previously described [10], V-ATPaseV1 became distributed on the perinuclear area, and the red fluorescence intensity of V-ATPaseV1 in treated cells was obviously weaker than that in control cells.

## 3. Discussion

The endocytosis pathway of LDL in cells regulates the systemic metabolite and homeostasis of cholesterol in vivo [22]. Surface charge is associate with the endocytosis of ENPs [2,3]. In this study, we fabricated a series of AuNPs (13.00 ± 0.69 nm) with varied surface-charge densities and found internalized AuNPs with HSNCD significantly reduced LDL uptake in HepG-2, HeLa, and SMMC-7721 cells compared with those in control cells.

Endocytosis is an important pathway to regulate the metabolism of LDL in cells. Davis et al. have demonstrated that LDL-R mutations blocking the release of LDL prevent receptor recycling, leading to LDL-R degradation in the lysosome [23], and the pathological accumulation of LDL in serum happens in the patient with the mutation. In other words, LDL uptake and degradation were regulated primarily by the varied abundance of LDL-R on the cell surface [11]. The abundance of LDL-R on the cell surface depends on not only gene expression [23] but also recycling of the LDL-R [1]. In this regard, our results suggest that AuNPs with HSNCD reduced LDL-R abundance on the cell surface; however, expression of LDL-R at the protein or mRNA level was not modulated by these nanoparticles. Therefore, it is reasonable to suggest that the reduction of LDL receptors on the cell surface was due to disruption of recycling of LDL-R because of these AuNPs.

In general, after dissociation of LDL-R from the LDL/LDL-R complex at endosome, the free LDL-R was transported to the cell surface by REs. We traced the transport of LDL-R and found, compared with the control cell, that more LDL-R was transported into the lysosome to degrade eventually.

To investigate the reason leading to the failure of LDL discharge from LDL-R, we determined the pH of endosomes. Our results showed that there were significantly fewer acidic endosomes in treated cells than in control cells, suggesting that the AuNPs disrupted pH regulation in endosomes by affecting activity of V-ATPase in endosomes. Further research revealed that disruption of the decrease in pH in EEs occurred because of the reduction in V-ATPaseV1 binding to V-ATPaseV0 on the endosome membrane, which could reduce the activity of V-ATPase to transport H^+^ ions from the cytoplasm to the endosomes; thus, the relatively high pH hampered the LDL-R release from LDL/LDL-R complex, thereby increasing the accumulation of the LDL/LDL-R complex at the EE, and less LDL-R was transported to the cell surface via the REs. These receptors were transported into the lysosomes to be degraded leading to a reduction in LDL-R on the cell surface. The effect of the AuNPs on the metabolism of LDL in vivo need further research.

## 4. Materials and Methods

### 4.1. Cell Culture and Treatment

Human cervical carcinoma (HeLa), human hepatoblastoma (HepG-2), and human hepatoma (SMMC-7721) cell lines were bought from National Infrastructure of Cell Line Resource and cultured in Dulbecco’s minimal essential media (DMEM)/high glucose supplemented with 10% fetal bovine serum (FBS) in a humidified atmosphere of 5% CO_2_ at 37 °C. In this study, medium A represents DMEM/high glucose containing 10% FBS, medium B represents DMEM/high glucose containing 10% lipoprotein-deficient fetal bovine serum (LPDS) (Millipore, Burlington, MA, USA), medium C is constituted with medium B and 10 μg/mL DiI-LDL (Luwen Biotech, Shanghai, China), respectively. Based on experiment requirements, cells seeded in a 6-well, 24-well, 96-well culture plate or confocal petri dish for 24 h. Then, the cells were subcultured in medium A containing AuNPs at an concentration of 3 μg/mL. After 24 h, discard the treatment medium and washed the cells with sterile phosphate buffer saline (PBS). The treated cells would be applied for further experiments.

### 4.2. AuNPs Synthesis

Synthesis of AuNPs spheres used citrate reduction as previously reported [24]. Briefly, we dissolved 1.2 mL 1% HAuCl_4_ solution in 100 mL deionized water, regulated PH to 3.42~3.46, heated to boil, and added 0.6 mL 0.5% sodium citrate, and kept stirring until solution color settled on wine-red. Collected the sediment after centrifuge at 7000× *g* for 20 min and stored in water at 4 °C in the dark. AuNPs stabilized by citrate (citrate–AuNPs) was obtained.

Surface-charge density regulated by the molar ratio of MUA and OT added [25]. 0.5 mg citrate–AuNPs was diluted in 20 mL reaction solution (0.25 mM sodium citrate solution with 0.05% Tween-20), regulated the PH to 10–11, argon was aerated for 5 min to clear the air, and 4 mM sulfhydryl compound (MUA, OT) 250 μL was added, and it was kept stirring for 16–18 h in seal tuber. We named the sample 0% MUA–AuNPs, 33% MUA–AuNPs, 50% MUA–AuNPs, 67% MUA–AuNPs, and 100% MUA–AuNPs based on the molar ratio of MUA and OT, i.e., 0:1, 1:2, 1:1, 2:1, and 1:0.

### 4.3. AuNPs Characterization

Absorption spectrum assayed using UV-Vis spectrometry (PERSEE, Shanghai, China). Hydrodynamic diameter and zeta potential were determined by DLS (Agilent, Santa Clara, CA, USA). TEM image was collected from a JEM 2100F microscope (JEOL, Tokyo, Japan) under 200 kV. The component elements of each element were detected by XPS (Beijing Synchrotron Radiation Facility, Beijing, China). Survey scans were run in the 0–720 eV range, while detailed scans were recorded for the Au 4f, C 1s, and S 2p regions.

### 4.4. LDL Uptake Assay

The treated cells were incubated in medium C for 2 h at 37 °C. We collected the cells and detected the MFI of the cells by FCM (BD Biosciences, Franklin Lakes, NJ, USA).

The treated cells on the confocal petri dish were reincubated in medium C for 2 h at 37 °C. We fixed the cells by 4% paraformaldehyde and stained nuclei with Hochest 33342 (Thermo Fisher Scientific, Waltham, MA, USA). Then, they were imaged with an LSCM (Nikon A1, Tokyo, Japan).

### 4.5. LDL-R on Cell Surface Assay

For detecting LDL binding, cells incubated in medium A contained AuNPs at 4 °C for 20 min. The treated cells were precooled at 4 °C and incubated in medium C at 4 °C for 20 min; then, we collected the cells and detected MFI by FCM. For detecting LDL-R abundance on cell surface, cells incubated in medium A contained AuNPs at 37 °C for 24 h. Then, we collected the cells and incubated with anti-LDL-R antibody with fluorescein isothiocyanate (FITC) (Abcam, Cambridge, UK) at 4 °C. After 1 h, we detected MFI of the cells by FCM.

For the imaging experiment, the treated cells on the confocal petri dish were incubated with medium C or medium B with LDL-R antibody with FITC for 20 min at 4 °C. We cleared unbound DiI-LDL or LDL-R antibody and observed the cells with LSCM.

### 4.6. Western Blot Assay

For LDL-R detection, the treated cells lysised by RIPA buffer containing 1 mM PSMF (Beyotime, Shanghai, China) at 4 °C for 20 min. Gathered the lysate and centrifuged at 14,000× *g* for 15 min. The protein concentration of the supernatant was detected by BCA kit (Beyotime), then boiled with SDS-PAGE loading buffer at 95 °C for 5 min and stored at –20 °C.

For V-ATPaseV1D detection, the treated cells were divided two equal parts. One part was treated as LDL-R detection. The other part was frozen in liquid nitrogen and thawed at room temperature several times. Then, we centrifuged the cells at 600× *g* for 5 min to remove the whole cells. Then, we centrifuged the supernatant at 1000× *g* for 10 min. Finally, the sediment was treated further as LDL-R detection.

20 μg protein samples were separated on SDS-PAGE gel and transferred to PVDF membrane. Immunoblotting was performed using primary antibodies of LDL-R, V-ATPaseV1D, and GAPDH at 4 °C for 16 h. Then, we incubated the PVDF membrane with the second antibody for 1 h at room temperature, imaged with automatic chemiluminescence imaging analysis system (Tanon, Shanghai, China) after the reactivated with enhanced chemiluminescent.

### 4.7. Reverse-Transcription PCR and Quantitative Real-Time PCR Analysis

Total RNA was extracted with Trizol reagent according to the manufacturer’s instructions (TianGen Biotech, Beijing, China). LDL receptor primer, foreword: ACCAACG AATGCTTGGACAAC; reverse: ACAGGCACTCGTAGCCGAT. Reverse transcription was performed as the instruction of the kit (TRANSGEN BIOTECH, Beijing, China). PCR amplification was detected by RT-PCR machine and Q-RT-PCR machine and normalized with GAPDH.

### 4.8. Immunofluorescence Assay

For LDL transporting pathway assay, the treated cells were incubated in medium C at 4 °C for 20 min; then, we removed the unbind DiI-LDL and recultured at 37 °C in medium A. After the indicated time, the cell were rapidly chilled to 4 °C to stop the internalization. The cells were fixed and incubated in blocking buffer (5% BSA and 0.3% Tween-20 in PBS) for 1 h at room temperature, then coincubated with primary antibody of Rab5) or LAMP1 for 16 h at 4 °C, then incubated with the second antibody (Abcam) for 2 h and stained nuclei with Hochest 33342. The treated cells were observed and imaged with LSCM.

For the LDL-R transporting pathway assay, the treated cells were fixed and incubated in blocking buffer for 1 h at room temperature, then coincubated with LDL-R antibody with FITC and primary antibody of LAMP1 for 16 h at 4 °C. The cells were treated further as LDL transporting pathway assay.

For V-ATPaseV1, we detected the subunit D. In briefly, one part of the treated cells were fixed directly, the other part were incubate with 0.003% digitonin at 4 °C for 15 min to release the V-ATPaseV1 in the cytoplasmic matrix before fixed by 100% methanol. The cells were treated further as LDL transporting pathway assay.

### 4.9. Endocytic pH Measurement

LysoSensor™ Green DND was used to measure the endocytic PH. The treated cells on confocal petri dish incubated in medium A contained 1 μm/mL lysosensor dye for 2 min at 37 °C. We replaced the medium with fresh medium A and observed the cells with LSCM at a magnification of 400×. We chose fifty cells to compute the MFI of a single cell. We used the MFI indicate the endocytic vesicles PH.

### 4.10. Statistical Analysis

Data were analyzed with SPSS ver. 19.0 software (SPSS, Chicago, IL, USA) using one-way analysis of variance (ANOVA). Results were validated by performing at least three independent experiments. The results were expressed as mean value ± standard error of the mean (SEM). Differences were considered significant at *p* < 0.05.

## 5. Conclusions

In this study, we revealed that AuNPs with HSNCD adjust the metabolism of LDL in cells. We demonstrated that the internalized AuNPs disrupted V-ATPaesV1 binding to V-ATPaesV0 on the vesicle membrane, thereby preventing the decrease in the pH of EEs, blocking the separation of LDL-R from LDL/LDL-R complex. The retained LDL-R could not be recycled to the cell surface and was eventually degraded in the lysosomes. This degradation consequently reduced abundance of the LDL-R on the cell surface, hampering metabolism of LDL by the endocytosis pathway of the receptor-mediated.

## Figures and Tables

**Table 1:** Zeta potential and hydrodynamic size of the AuNPs in medium.

Mixed Charged Thiols (11-mercaptoundecanoic acid (MUA)/1-octanethiol (OT))	Zeta Potential (mV) (Mean ± SEM)	Hydrodynamic Size (nm) (Mean ± SEM)
0:1 (0% MUA–AuNPs)	−20.21 ± 0.48	42.12 ± 1.32
1:2 (33% MUA–AuNPs)	−19.92 ± 0.43	42.23 ± 2.12
1:1 (50% MUA–AuNPs)	−16.06 ± 0.38	43.14 ± 1.24
2:1 (67% MUA–AuNPs)	−14.54 ± 0.52	42.67 ± 1.52
1:0 (100% MUA–AuNPs)	−33.14 ± 0.42	42.45 ± 2.38
Citrate–AuNPs	−32.05 ± 1.77	41.5 ± 2.42

Error ranges represent standard error of the mean (SEM).

## Supplementary Materials

The following are available online at http://www.mdpi.com/1422-0067/19/9/2790/s1.
