# Supplementary material for: Nanoparticles with High-Surface Negative-Charge Density Disturb the Metabolism of Low-Density Lipoprotein in Cells
Source: Int J Mol Sci. 2018 Sep 17;19(9):2790. doi: 10.3390/ijms19092790 (PMC6164102; doi:10.3390/ijms19092790)
Supplement: Supplementary file 1 [file ijms-19-02790-s001.pdf]

Supplementary Information

Supplemental methods

AuNPs internalized by cell

After the incubation with AuNPs for 24 h at 37℃, the cells were collected and counted the number by cytometry. The cells were dissolved in aqua regia for 24 h and re-dissolved in solution with 1% HCl and 3% HNO_3. The samples were detected by Inductively Coupled Plasma Mass Spectrometry (ICP-MS) (Thermo Fisher Scientific, USA).

Cytotoxicity Assessment

Cell Counting - 8 Kit (Dojindo Laboratories, Japan) was carried out to measured the cell viability after AuNPs treatment. Cell cultured in 96-well plates with 0.5×10^4 cells per well for 24 h, then replace the medium with fresh medium containing 1, 3, 6 and 30 μg/ml gold nanoparticles (AuNPs) with 8-well replicates. After 24 h, take away the supernatant, rinsed with sterile PBS and added the compete medium contain 10% CCK-8 solution. After 40 min, detect the absorbance at wavelength 450 nm using a microplate reader (SpectraMax M5, Molecular Devices, USA). Cell apoptosis was measured by Annexin V-FITC/PI Kit (Dojindo Laboratories, Japan) according to the manufacturer's instruction. In brief, collected the treated cells with 0.25% trypsin and washed the cell for two times by sterile PBS and add the stain buffer contain PI and Annexin V-FITC for 15 min. Detect the mean fluorescence intensity (MFI) use flow cytometry（FCM）(Accuri C6, BD, USA)

Supplemental Results

AuNPs characterization

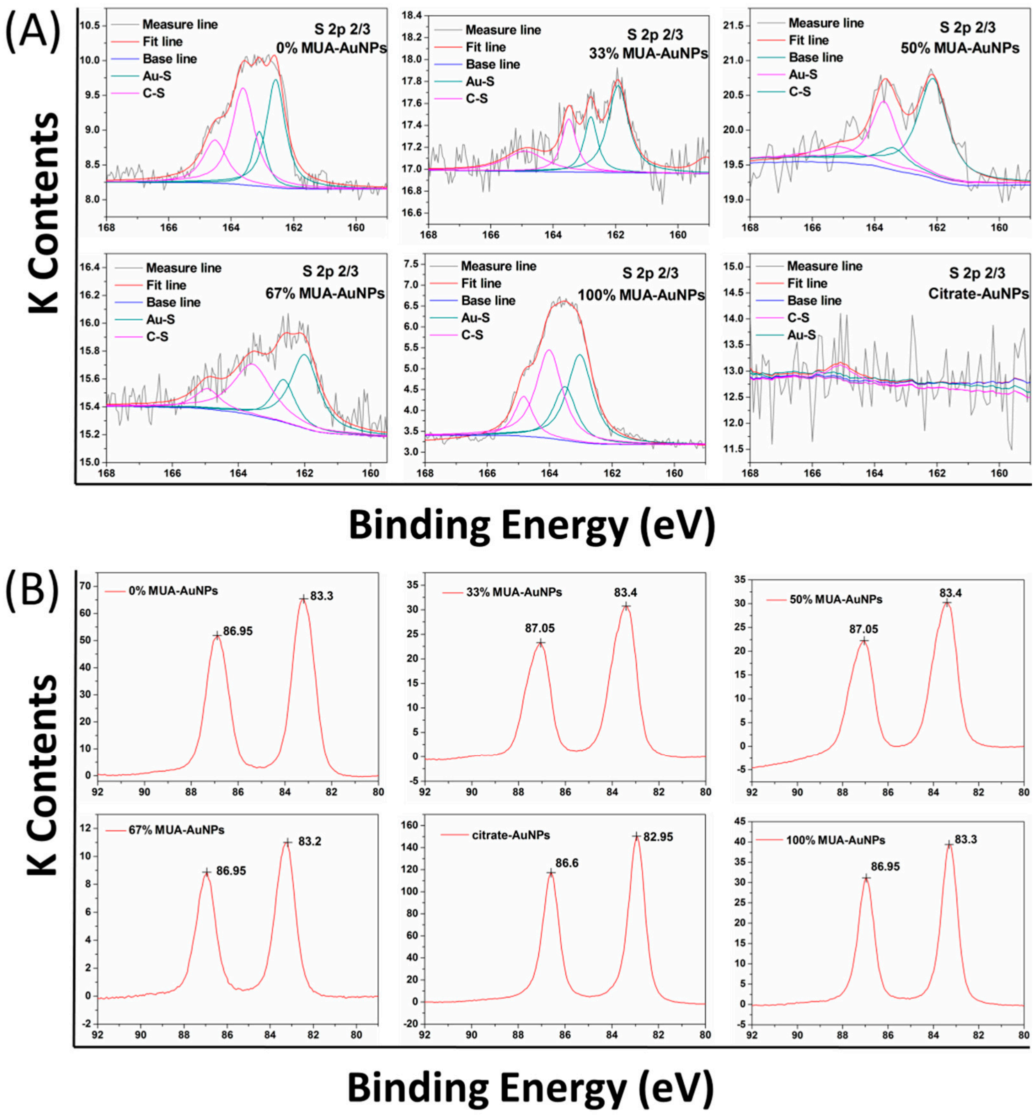

Figure S1. XPS spectrum of S 2p doublet (A) and Au4f doublet (B).

AuNPs internalized by cell

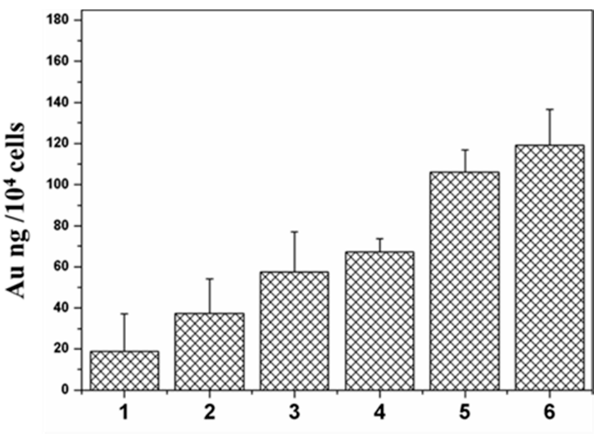

Figure S2. AuNPs uptake by HepG-2 cell measured by ICP-MS. 1, 2, 3, 4, 5, 6 in figure represent 0% MUA-AuNPs, 33% MUA-AuNPs, 50% MUA-AuNPs, 67% MUA-AuNPs, 100% MUA-AuNPs and citrate-AuNPs.

Cytotoxicity Assessment of AuNPs

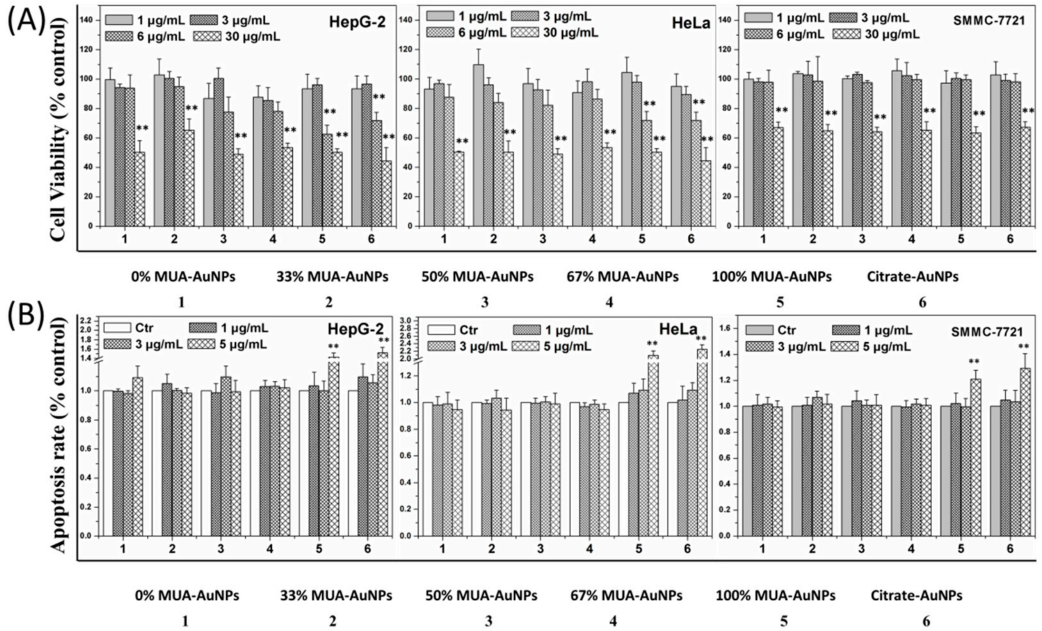

Figure S3. Cell viability evaluated by CCK-8 (A) and apoptosis ratio detected by Annexin-V FITC/PI Kit (B). 1, 2, 3, 4, 5, 6 in figure represent 0% MUA-AuNPs, 33% MUA-AuNPs, 50% MUA-AuNPs, 67% MUA-AuNPs, 100% MUA-AuNPs and Citrate-AuNPs. The statistical data

represents the mean ± SEM values from our three independent experiment.

Effects of AuNPs on recycling of LDL-R in cells

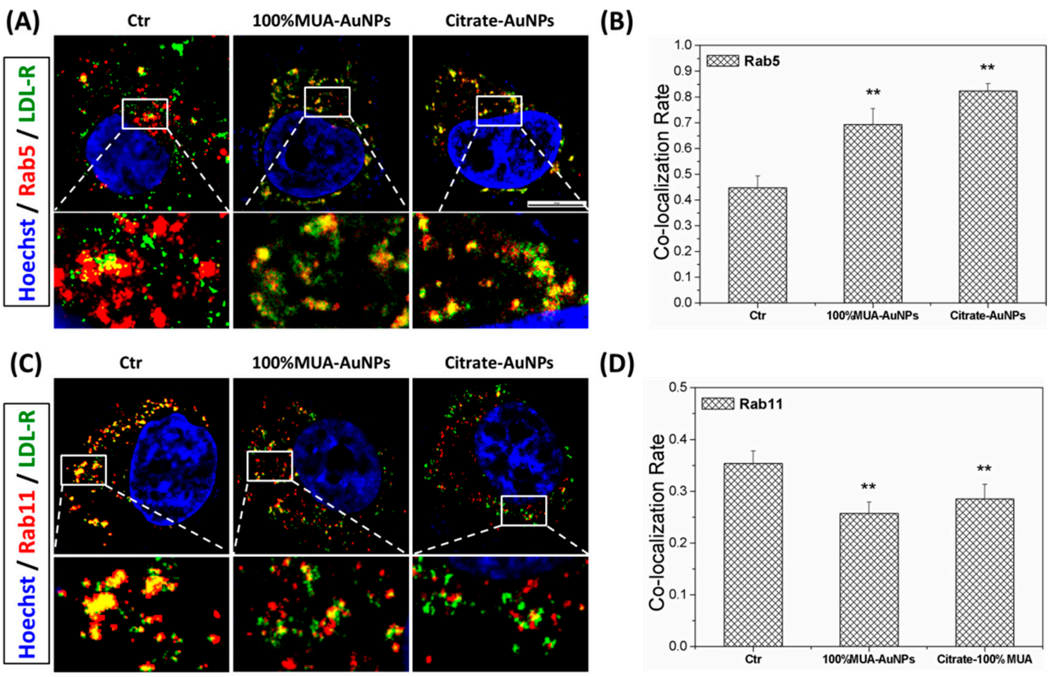

Figure S4. Effects of AuNPs on the LDL-R distribution in cells. The co-localization of LDL-R with EEs (A) and REs (C). The co-localization rate of LDL-R in EEs (B) and REs (D). The co-localization rates are presented as the means ± standard deviations from three independent experiments; more than 30 cells were evaluated for each experiment. $^{**}p < 0.01$, $^{*}p < 0.05$ indicate significant differences compared with the control group. Bar = 10 μm.
